# Haplotypes in *IL-8* Gene Are Associated to Age-Related Macular Degeneration: A Case-Control Study

**DOI:** 10.1371/journal.pone.0066978

**Published:** 2013-06-19

**Authors:** Federico Ricci, Giovanni Staurenghi, Tiziana Lepre, Filippo Missiroli, Stefania Zampatti, Raffaella Cascella, Paola Borgiani, Luigi Tonino Marsella, Chiara Maria Eandi, Andrea Cusumano, Giuseppe Novelli, Emiliano Giardina

**Affiliations:** 1 UOSD Patologia retinica Fondazione PTV “Policlinico Tor Vergata”, Rome, Italy; 2 Eye Clinic, Department of Clinical Science “Luigi Sacco”, Sacco Hospital, University of Milan, Milan, Italy; 3 Department of Biomedicine and Prevention, Centre of Excellence for Genomic Risk Assessment in Multifactorial and Complex Diseases, School of Medicine, University of Rome “Tor Vergata”, Rome, Italy; 4 Department of Clinical Physiopathology, Eye Clinic, University of Torino, Turin, Italy; 5 National Agency of Evaluation of Universities and Research (ANVUR), Rome, Italy; 6 S. Pietro Fatebenefratelli Hospital, Rome, Italy; University of Tor Vergata, Italy

## Abstract

**Background:**

Age-related macular degeneration (AMD) is the main cause of blindness in the developed world. The etiology of AMD is multifactorial due to the interaction between genetic and environmental factors. IL-8 has a role in inflammation and angiogenesis; we report the genetic characterization of IL-8 allele architecture and evaluate the role of SNPs or haplotypes in the susceptibility to wet AMD, case-control study.

**Methods:**

Case-control study including 721 AMD patients and 660 controls becoming from Italian population. Genotyping was carried out by Real Time-PCR. Differences in the frequencies were estimated by the chi-square test. Direct sequencing was carried out by capillary electrophoresis trough ABI3130xl.

**Results:**

rs2227306 showed a p–value of 4.15*10^−5^ and an Odds Ratio (OR) for T allele of 1.39 [1.19–1.62]. After these positive results, we sequenced the entire *IL-8* regulatory and coding regions of 60 patients and 30 controls stratified for their genotype at rs2227306. We defined two different haplotypes involving rs4073 (A/T), rs2227306 (C/T), rs2227346 (C/T) and rs1126647 (A/T): A-T-T-T (*p-value*: 2.08*10^−9^; OR: 1.68 [1.43–1.97]) and T-C-C-A (*p-value*: 7.07*10^−11^; OR: 0.60 [0.51–0.70]). To further investigate a potential functional role of associated haplotypes, we performed an expression study on RNA extracted from whole blood of 75 donors to verify a possible direct correlation between haplotype and gene expression, failing to reveal significant differences.

**Conclusions:**

These results suggest a possible secondary role of *IL-8* gene in the development of the disease. This paper outlines the importance of association between inflammation and AMD. Moreover IL-8 is a new susceptibility genomic biomarker of AMD.

## Introduction

Age-related macular degeneration (AMD), the main cause of blindness in the developed world, affects more than one million Italian people. The etiology of AMD is multifactorial due to the interaction between genetic and environmental factors. As with other complex diseases, recent genome-wide studies have established and confirmed many genetic variants associated with higher risk for AMD.[1], [2] Genetic and functional studies have confirmed that inflammation plays a pivotal role in the pathogenesis and progression of AMD. Above all, C-reactive protein has been associated with AMD [3], [4] as well as different interleukin genes, including *IL-*6 [3] and *IL-8*. [5], [6] Despite this, the genomics of certain interleukin pathways in AMD is poorly understood, probably due to their small effect on overall susceptibility, which may lead to false negative findings in genome-wide association studies (GWAS). Interleukins mediate many of the effector phases of immune and inflammatory responses. In addition, activated macrophages secrete many proteolysis enzymes, such as collagenases and elastases, which may fragment Bruch's membrane and promote the neovascularization typical of wet AMD. The interindividual variability, in terms of quality and quantity of cytokines produced, is associated with single nucleotide polymorphisms (SNPs) and copy number variations (CNVs) involving promoters, introns, exons, and regulatory elements. [7]–[9] Interleukin-8, a member of the CXC chemokine family, is a chemoattractant of neutrophils and lymphocytes. *IL-8* encodes a protein that mediates effects by interacting with two cell-surface G protein-coupled receptors, CXCR1 and CXCR2. [10], [11] A wide variety of cells can express IL-8 with the principal role of initiating and amplifying acute inflammatory reactions.[12] Additionally, IL-8 plays a pivotal role in cancer pathogenesis, including angiogenesis, tumor growth, and metastasis.[13] Despite its role in inflammation and angiogenesis, little is known about the effects of *IL-8* genetic variants in the development of wet AMD.[6] Here, we report the genetic characterization of IL-8 allele architecture and evaluate the role of SNPs or haplotypes in the susceptibility to wet AMD through a well-powered, case-control study.

## Materials and Methods

### Ethics Statement

The study was conducted according to the Declaration of Helsinki, and informed consent was obtained from the participating subjects after the nature of the study had been explained. The study protocol was approved by the ethics committee of the University of Rome Tor Vergata, Rome, Italy.

### Patients' recruitment

The 1381 Italian subjects recruited for this study were classified in two different groups: patients diagnosed with exudative AMD (case group, n = 721) and subjects clinically evaluated as not having any form of macular degeneration (control group, n = 660). The Medical Retina Centre of Policlinico of Tor Vergata in Rome provided a thorough clinical evaluation of case and control groups, and all subjects were included in the study according to criteria explained in Ricci et al.[14] The same inclusion criteria were used from the other centre involved in our study: the Eye Clinic, Department of Clinical Science “Luigi Sacco” in Milan and the Department of Clinical Physiopathology, Eye Clinic, University of Torino. Clinical data collected were age at diagnosis, bilaterality, family history, and CNV (choroidal neovascularization) type (Table 1). Furthermore a questionnaire on environmental factors was submitted to patients and controls recruited to assess their smoke habits. All patients and controls were sex matched.

**Table 1:** Clinical characteristics of samples tested.

Characteristic	Patients with AMD (n:721)	Healthy control subjects (n:660)
Age, year: range	52–91	53–88
Age, year: mean (SD)	75.0 (7.6)	69.2 (11.1)
Sex: man (%)	324 (45)	304 (46)
Sex: woman (%)	397 (55)	356 (54)
Family history of AMD (%)	397 (55)	NA
CNV: classic (%)	325 (45)	NA
CNV: occult (%)	396 (55)	NA
Bilatelar neovascular AMD (%)	72 (10)	NA
Age at diagnosis, mean (SD)	74.3 (7.9)	NA
Smoke habits: never	362 (50.2)	516 (78.2)
Smoke habits: ex smoker	213 (29.6)	NA
Smoke habits: smoker	146 (20.2)	144 (21.8)

NA: not applicable.

### Genetic analysis

#### Genotyping

We first evaluated the association of an intronic SNP in the *IL-8* gene: rs2227306. Genotyping was performed by TaqMan assays (Applied Biosystems, Foster City, California). Reactions were run in an AB7500 Fast real-time PCR machine (Applied Biosystems) and interpreted using Sequence Detection System 2.1 software (Applied Biosystems). Each plate contained three positive control samples previously confirmed by direct sequencing and a negative control sample. No departure from Hardy-Weinberg equilibrium was detected. Results from genotype assessment were confirmed by direct sequencing of 10 random samples.

#### Sequencing

We sequenced the entire genomic sequence of *IL-8*, including the promoter region, in 60 cases and 30 controls. Direct sequencing was performed through seven different amplicons: one for the promoter, one for exon 1, and five for exon 2 (Table 2). PCR was performed using AmpliTaq Gold DNA Polymerase (Applied Biosystems) according to the manufacturer's instructions. PCR products were purified, and PCR sequence reactions were performed with BigDye Terminator v3.1. After purification with BigDyeXTerminator (Applied Biosystems), samples were run on an ABI3130xl (Applied Biosystems). We also sequenced the entire genomic sequence and the cDNA sequence of the 75 subjects recruited for the expression study (see Table 2 for primers).

**Table 2:** Primers used for *IL8* sequencing.

*IL8* region	Forward Primer (5′-3′)	Reverse Primer (5′-3′)	Length of amplicon	Annealing Temperature
Promoter	GACAAGTACCTAGTCTTATC	CTGAAAGTTTGTGCCTTATG	685	56°
Exon1	AGTGTGATGACTCAGGTTTG	GAATAGCTTTGCTATCTAAGG	368	58°
Exon2	AGGAAGTAGCTGGCAGAGCT	TTCTCCACAACCCTCTGCAC	545	62°
	CTTTCTGATGGAAGAGAGCT	TATTGCATCTGGCAACCCTA	627	58°
	GATGCCAGTGAAACTTCAAG	TCACAACATCACTGTGAGGT	543	58°
	AAGTCCTTGTTCCACTGTGC	TGACTGTGGAGTTTTGGCTG	546	60°
	TCCTAGTTTGATACTCCCAG	GCTAAGGGGAAGCATTTATG	425	58°
cDNA	ACAAGCTTCTAGGACAAGAG	ATGAATTCTCAGCCCTCTTC	315	58°

### Expression study

#### Real-time PCR

The generation of first-strand cDNA was performed using a high capacity DNA reverse transcription kit (Applied Biosystems) according to the manufacturer's instructions. The reverse transcriptase reaction products were used for quantitative real-time PCR, which was run in an AB7500 (Applied Biosystems) using TaqMan predesigned probes (Hs00174103_m1). The amount of *IL-8* mRNA present in each sample was normalized to the amount of mRNA of the *HPRT* reference gene in the same sample. Relative mRNA expression levels of all examined genes were measured using the comparative 2^−ΔΔCT^ method. Each plate contained a negative control.

### Statistical analysis

Statistical analyses were performed by a standard 2×2 table and Fisher's exact tests. Allele and genotype frequencies of all the variations tested were compared with those of the clinically examined control subjects (n = 660). Allele analysis in unrelated samples was performed using the software UNPHASED [15]. The ORs were calculated by the online software “Calculator for Confidence Intervals of Odds Ratio in an Unmatched Case Control Study” [16]. The binary logistic regression has been carried out by SPSS Program (SSPS Inc., IL, USA).

## Results

We tested the association between rs2227306 and wet AMD in a discovery cohort composed of 362 cases and 660 controls (Table 3). We obtained a significant p-value when we compared allele frequencies between cases and controls (*p* = 0.001). The T allele of rs2227306 achieved an odds ratio (OR) of 1.38 (95% CI = 1.14–1.67). We confirmed these findings in a replication cohort of patients (n = 359) and obtained the same level of association (*p* = 0.001) and a comparable OR (1.40; 95% CI = 1.16–1.69). In the merged cluster of samples, composed of both discovery and replication cohorts (n = 721), we obtained a p-value of 4.15×10^−5^ and an OR of 1.39 (95% CI = 1.19–1.62) for the T allele. As expected, genotype association confirmed these findings (Table 4). No differences in allele frequencies were detected after phenotype stratification (classic vs occult cnv, data not shown).

**Table 3:** Allelic association of rs2227306.

Cohort	Risk allele	AMD (Counts and frequency) (n:721)	Controls (Counts and frequency) (n: 660)	*P*	OR (95% CI)
I: n:362	T	281 (0.39)	416 (0.08)	0.001	1.38 (1.14–1.67)
II: n:359	T	281 (0.39)	416 (0.08)	0.001	1.40 (1.16–1.69)
I + II: n:721	T	562 (0.39)	416 (0.08)	4.15*10^−5^	1.39 (1.19–1.62)

**Table 4:** Genotypic association of rs2227306.

Cohort	Genotype	AMD (Counts and frequency) (n:721)	Controls (Counts and frequency) (n: 660)	*P*	OR (95% CI)
I: n:362	CC	142 (0.39)	308 (0.46)	0.002	0.74 (0.57–0.97)
	CT	159 (0.44)	288 (0.44)		1.20 (0.91–1.58)
	TT	61 (0.17)	64 (0.10)		2.07 (1.38–3.10)
II: n: 359	CC	133 (0.37)	308 (0.46)	0.002	0.67 (0.52–0.88)
	CT	171 (0.48)	288 (0.44)		1.38 (1.04–1.81)
	TT	55 (0.15)	64 (0.10)		1.99 (1.32–3.01)
I + II: n: 721	CC	275 (0.38)	308 (0.46)	3.55*10^−5^	0.70 (0.57–0.87)
	CT	330 (0.46)	288 (0.44)		1.28 (1.02–1.61)
	TT	116 (0.16)	64 (0.10)		2.03 (1.44–2.87)

On the basis of these positive preliminary results, we sequenced the entire *IL-8* regulatory and coding regions to understand the allele architecture of the gene and to identify causative SNPs or haplotypes. From 90 samples, including 20 cases for each genotypic class of rs2227306 and 30 controls, we detected two different conserved genomic regions in subjects carrying the T or C allele of rs2227306. The conserved regions involved three different SNPs: rs2227306 (C/T), rs2227346 (C/T), and rs1126647 (A/T). Complete linkage disequilibrium (D′ = 1) was detected between each SNP. Through sequencing, we defined two haplotypes: CCA and TTT. The associated haplotypes cover the promoter of a gene tagged by the marker rs4073 (A/T), although some recombination has been observed in both cases and controls (5% and 4%, respectively; Table 5). The small fraction of recombination makes it difficult to obtain a clear interpretation of recombinant haplotypes to disclose or exclude causative effects of single SNPs. To further investigate a potential functional role of associated haplotypes, we performed an expression study on RNA extracted from whole blood of 75 donors to verify the existence of a direct correlation between haplotype and gene expression. Of the tested samples, 25 were homozygous for haplotype T-C-C-A, 25 were homozygous for haplotype A-T-T-T, and 25 were heterozygous for rs2227306. We failed to detect differences in the expression of *IL-8* among these different haplotype and genotype classes. To investigate a potential role of the associated haplotypes on splicing and/or post-trascriptional regulation we also resequenced the cDNA of these subjects but failed to reveal any sequence differences.

**Table 5:** Frequencies and association of haplotypes involving rs4073 (A/T), rs2227306 (C/T), rs2227346 (C/T) and rs1126647 (A/T).

Haplotypes	Cases frequencies	Control frequencies	P	OR (95% CI)
A-C-C-A	0.05	0	NA	NA
A-T-T-T	0.39	0.28	2.08*10^−9^	1.68 (1.43–1.97)
T-C-C-A	0.56	0.68	7.07*10^−11^	0.60 (0.51–0.70)
T-T-T-T	0	0.04	NA	NA

NA: not applicable.

We performed a binary logistic regression considering the two haplotypes (T-C-C-A and A-T-T-T) and the smoke habit as independent variables and the status case/control as dependent variables. The contribution of smoke is statistically significant (p<0.0001) showing an OR[Exp(B)] of 3.424 (CI 95% 2.036–5.758). The multivariate logistic regression was carried out also to investigate the impact of age on the disease. Despite the significant association between age and disease (p = 0.001), the OR[Exp(B)] was 1.062 (CI 95% 1.024–1.102).

## Discussion

In this study, we identified two haplotypes associated with the development of AMD and confirmed these findings in two independent sample cohorts, including 721 patients and 660 controls. Functional studies failed to reveal a role of these haplotypes on mRNA expression and splicing. Taken together, these results confirm a role for *IL-8* in AMD pathogenesis. Despite its role in inflammation and angiogenesis, little is known about the effects of *IL-8* genetic variants in the development of AMD and further studies are needed to better understand its biological role in the pathogenesis of the disease. [17]–[20] It is well known that most of visual loss occurs in the late stages of the disease due to one of two processes: neovascular (wet) age-related macular degeneration and geographic atrophy (“late dry”). Genetic background of AMD confirm these aspects as the large majority of genes associated to its development are involved in inflammation regulation, most notably complement Factor H.[21] The *IL-8* gene, located on chromosome 4q12–q13, encodes for the most potent chemokine known and responsible for inducing chemotaxis, which is the directed migration of cells to a site of inflammation.[22] This chemokine is important in the regulation of the inflammatory response for its ability to recruit and activate acute inflammatory cells. It is also able to mediate the activation and migration of neutrophil also involved in the pathogenesis of disease. [23].

It is notably that in human coronary atherosclerosis, IL-8 is an important mediator of angiogenesis and may contribute to plaque formation via its angiogenic properties. [24] In this context IL-8 could represent a candidate gene to link the development and progression of AMD, the smoke habits and the occurrence of cardiovascular events in these patients. Oxidative stress has been shown to promote IL-8 production in several cell types. ROS (reactive oxygen species) can activate transduction pathways such as mitogen-activated protein kinases that contribute to the production of pro-inflammatory cytokines such as (IL)-8. The dysregulation of IL-8 balance can lead to the type of serious tissue damage that has been reported in several diseases such IBD (Inflammatory Bowel Disease), severe asthma and in patients with active colitis [25]–[26].

Oxidative stress and inflammation are interrelated. Whereas oxidative stress triggers inflammatory responses and inflammation also enhances the production of reactive oxygen species. It is to notice that recent works indicate that oxidative inactivation of the proteasome is a mechanistic link between oxidative stress and increased production of IL-8 in cultured RPE. [27]–[29] While further studies will be needed to assess the pathogenic role of IL8 and its receptors in the development of AMD, the strength of genetic association of IL8 here reported, suggest that *IL-8* SNPs should be included in the subset of variations to be tested to calculate individual risk for developing AMD. [30]–[34].
