# Correction: Haplotypes in *IL-8* Gene Are Associated to Age-Related Macular Degeneration: A Case-Control Study

**DOI:** 10.1371/annotation/d8311dd7-2499-4ef1-b731-b4830b1612df

**Published:** 2013-07-10

**Authors:** Federico Ricci, Giovanni Staurenghi, Tiziana Lepre, Filippo Missiroli, Stefania Zampatti, Raffaella Cascella, Paola Borgiani, Luigi Tonino Marsella, Chiara Maria Eandi, Andrea Cusumano, Giuseppe Novelli, Emiliano Giardina

Author Francesco Viola was omitted from the author byline. The full, correct list of authors and affiliations is as follows:

Federico Ricci^1^

Giovanni Staurenghi^2^

Tiziana Lepre^3^

Filippo Missiroli^1^

Stefania Zampatti^3^

Raffaella Cascella^3^

Paola Borgiani^3^

Luigi Tonino Marsella^3^

Chiara Maria Eandi^4^

Francesco Viola^7^

Andrea Cusumano^1^

Giuseppe Novelli^5,6^

Emiliano Giardina^3^

**1** UOSD Patologia retinica Fondazione PTV "Policlinico Tor Vergata", Rome, Italy

**2** Eye Clinic, Department of Clinical Science "Luigi Sacco", Sacco Hospital, University of

Milan, Milan, Italy

**3** Department of Biomedicine and Prevention, Centre of Excellence for Genomic Risk Assessment in Multifactorial and Complex Diseases, School of Medicine, University of Rome "Tor Vergata", Rome, Italy

**4** Department of Clinical Physiopathology, Eye Clinic, University of Torino, Turin, Italy

**5** National Agency of Evaluation of Universities and Research (ANVUR), Rome, Italy

**6** S. Pietro Fatebenefratelli Hospital, Rome, Italy

**7** UO Oculistica Fondazione IRCCS Cà Granda Ospedale Maggiore Policlinico, University of Milan, Milan, Italy 

